# Comparison of Photothermal and Photodynamic Diode Laser Therapy in Patients with Peri-implant Mucositis: A Systematic Review

**DOI:** 10.4317/jced.60711

**Published:** 2023-09-01

**Authors:** Rebeca Sánchez-Martos, Naomi-Abawa Kronkah, Santiago Arias-Herrera

**Affiliations:** 1 Universidad Europea de Valencia. Faculty of Health Sciences. Department of Dentistry

## Abstract

**Background:**

To determine whether photodynamic laser therapy or photothermal laser therapy demonstrates greater improvements in the clinical signs of peri-implant mucositis as an adjuvant to mechanical debridement.

**Material and Methods:**

Electronic databases were used to select articles on February 10th, 2022. The clinical outcomes analysed were the plaque index (PI), probing depth (PD) and bleeding of probing index (BoP). The following PICO question was formulated: Among patients with peri-implant mucositis, does photothermal laser therapy (PT) demonstrate greater improvement in clinical inflammatory signs in comparison to antimicrobial photodynamic therapy (aPDT) as an adjuvant to conventional therapy?

**Results:**

Seven randomized controlled trials (RCTs) were included in the systematic review. The clinical parameters were compared amongst all studies at baseline and 3-month follow-up appointment. aPDT reduced both PI and PD great than PT. PT showed greater reductions in BoP.

**Conclusions:**

Conclusions are difficult to generalize due to the heterogeneity in the methodology of the included studies. However, this systematic review suggests that aPDT alongside mechanical debridement demonstrated greater improvements in the PI and PD. Other factors besides the laser therapy itself may account for these findings. As for BoP index, PT demonstrated greater improvements due to its photo-biomodulating effects. Clinical Relevance: In patients with peri-implant mucositis, the combination of photothermal diode laser therapy and mechanical debridement entails promising results in treating and preventing the progression of the pathology.

** Key words:**Peri-implant mucositis, Photothermal diode laser therapy, Photodynamic diode laser therapy, Bleeding on probing.

## Introduction

Dating back to prehistoric times, anthropologists and palaeontologists have brought forth several findings demonstrating human beings practicing dental replacement. The loss of tooth structure has been problematic since the dawn of humanity mainly because of one’s inability to sustain oneself. Henceforth, dental replacements have evolved tremendously into what is now known as modern dental implantology. Considering that there is an increasing number of patients seeking dental implant treatments, the prevention and treatment of their associated complications illustrate a serious and relevant challenge (1).

The most frequent complication that may arise subsequent to implant placement is peri-implant mucositis. Based on a 2021 cross-sectional study carried out by Romandini *et al*., the prevalence of peri-implant mucositis is 31.9% (2). According to the most recent consensus report from the World Workshop in Periodontology, peri-implant mucositis is defined as a reversible peri-implant mucosal inflammation in absence of continuous marginal peri-implant bone loss (3). Peri-implant mucositis is an unfavourable condition that arises due to the pathological transformation of healthy peri-implant mucosal tissue to one that is pathogenic. Notably, the surfaces of the titanium dental implant acquire a bacterial biofilm which then initiates an inflammatory response.

 Although the accumulation of pathogenic bacterial on the biofilm is the main risk factor with the most scientific evidence involved in the development of peri-implant mucositis, other risk factors associated with this pathology have been documented as well. Some of the evidence-based risk factors include deficient oral hygiene, tobacco consummation and previous history of periodontitis or mucosal diseases. Furthermore, the absence of keratinized mucosa influences hygiene levels and the health of peri-implant tissue causing its retraction (4). The main clinical manifestation and key diagnostic factor is bleeding on gentle probing. Other signs and symptoms include erythema, swelling and/or suppuration (3).

Due to its reversibility, it is important to stress prophylactic measures, early diagnosis, as well as prompt treatment in order to prevent the evolution of peri-implant mucositis into a much more aggressive pathology: peri-implantitis. Currently, the most widely used treatment for peri-implant mucositis is to perform a non-surgical approach based on mechanical debridement, however, it has been observed that the bacterial load returns to baseline levels after 3 months (5). Complete destruction of bacteria is difficult to achieve with conventional therapy alone (4). This limiting outcome has been depicted in multiple studies including that carried out by Salvi *et al*. Their study demonstrated that despite mechanical debridement showing a reduction of gingival inflammation, there was still an elevated level of inflammatory host markers such as matrix-metalloproteinase-8 (6). Due to these limitations, adjuvant elements are being studied to improve clinical outcomes. One of the most studied therapies today is the use of diode laser for phototherapy purposes. The main photobiological effects of periodontal phototherapy are photothermal and photochemical effects.

Photothermal therapy (PT) functions due to an increase in local temperature induced by the action of the laser. The light energy is exposed to the tissue for a period inducing a thermal interaction. Lasers’ photobiomodulation characteristic depends heavily on the amount of energy applied. Low-level laser therapy (LLLT) promotes cellular regeneration without producing irreversible thermal changes (7). PT is beneficial due to its microbial decontaminating and bio-stimulating effects.

Antimicrobial Photodynamic therapy (aPDT) is a laser therapy based on a photochemical mechanism of action. It involves the use of a photosensitizer, laser light source and tissue molecular oxygen. A pigment called a photosensitizer is used to selectively reach the targeted cell or microorganism aimed to be eliminated. In essence, photosensitizers are exposed to a light source at a wavelength specific to the selected pigment. This results in the photosensitizer to become energized to what is known as a highly energized triplet-state. These energized photosensitizer molecules are then ultimately exposed to tissue oxygen in order to cause cellular damage (7,8). This therapy can successfully kill bacteria, fungi, viruses, and resistant microbes without altering the surfaces of implants.

Currently, there are no systematic reviews comparing the efficacy of PT to aPDT in combination with mechanical debridement. Therefore, the purpose of this systematic review is to determine which laser therapy, PT or aPDT, demonstrates greater improvement in clinical signs of peri-implant mucositis as an adjunct to mechanical debridement through the evaluation of the plaque index, probing depth, and bleeding on probing index.

## Material and Methods

-Protocol and focused question

The Preferred Reporting Items for Systematic Review and Meta- Analysis (PRISMA) guideline was followed to perform this systematic review (9). The following clinical question was formulated based on the PICO structure: Among patients with peri-implant mucositis (P), does photothermic laser therapy (I) demonstrate greater improvement in clinical inflammatory signs (O) in comparison to photodynamic therapy (I) as an adjuvant to conventional therapy (C)?

-Selection criteria.

Studies were included based on the following inclusion criteria: 1) cohort study or randomized control trial (RCT); 2) population based on patients with peri-implant mucositis; 3) intervention used either PT diode laser therapy or aPDT diode laser therapy as an adjuvant to conventional therapy; 4) clinical outcome measured includes the bleeding on probing index; 5) follow-up of at least 3 months. Studies were excluded based on the following exclusion criteria: 1) animal and in-vitro studies; 2) studies published in 2011 or before; 3) studies in languages other than English or Spanish.

**Figure 1:**
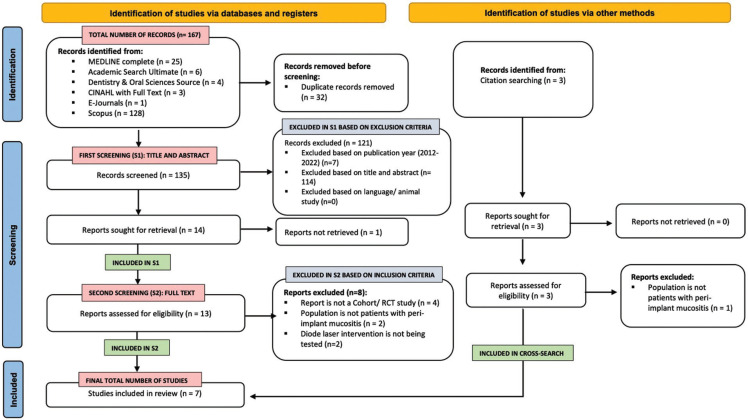
Study identification process and results of the literature search via databases and other methods according to PRISMA 2020.

-Search strategy:

Both CRAI library Ducle Chacón and Elsevier’s Scopus search engines were used to perform the search on February 10th, 2022. The databases included can be seen in Fig. 1. Keywords and Medical Subject Heading (MeSH) terms were used to construct the following search algorithm: (“Peri-implant mucositis” OR “Peri-implant disease” OR “Mucositis”) AND (“Photothermic” OR “Photodynamic” OR “Diode laser” OR “Laser Therapy” OR “Photothermal Therapy” OR “Phototherapy” OR “Laser, Semiconductor/ therapeutic use” OR “Photochemotherapy”) AND (“Conventional therapy” OR “Conventional non-surgical therapy” OR “Mechanical debridement” OR “Mechanical curettage” OR “Periodontal debridement” OR “Dental Scaling” OR “Dental prophylaxis”) AND ( “Clinical inflammatory signs” OR “Plaque index” OR “bleeding on probing index” OR “Gingival Index”).

-Screening methods and data abstraction.

Two impartial reviewers (NK and RS) independently performed the systematic review search. Once the duplicates between the two databases had been eliminated, two screening phases were performed to determine the eligibility of the studies. The first screening phase consisted of selecting relevant articles based on their title and abstract. Relevant articles were then excluded based on the exclusion criteria. The remaining articles were therefore the total number of articles included after the first screening phase. The second screening phase consisted of reading the full texts of the articles included in the first screening phase. Articles were then excluded if they do not fit the inclusion criteria. The bibliography of each article was then reviewed to perform a cross-search. Outside resources were also employed. Relevant studies were first selected based on their title and abstract. The full text was then read completely and only those satisfying the eligibility criteria were selected. The remaining articles were therefore the total number of articles included after performing a cross-search.

The studies selected in the second search phase and cross-search were included in the systematic review. Any disagreement in study eligibility was resolved by discussion between both reviewers until a consensus was reached. The level of agreement between the reviewers was calculated using the k-score according to the Landis & Koch criteria (10).

**Figure 2:**
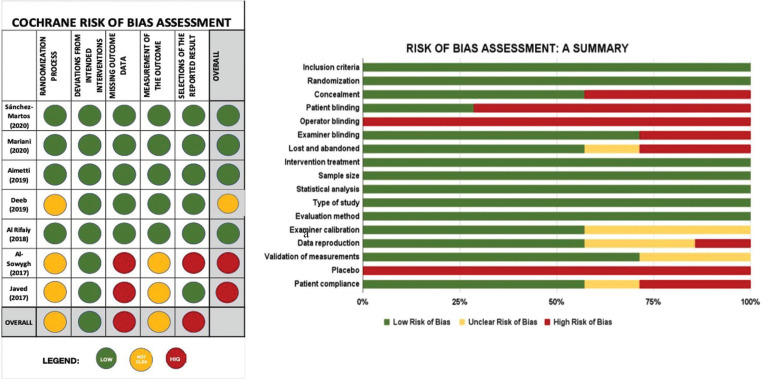
a. Risk of bias according to the Cochrane system. b. Risk of bias summary, review authors’ judgments about each risk of bias item presented as percentages across all included studies.

-Risk of bias in individual studies:

The risk of bias was assessed independently by the same reviewers who performed the search (NK and RS) according to the Cochrane collaboration’s tool shown in Fig. 2. (11). Other sources of bias, seen in Fig. 2, were also recorded.

-Case definitions

Peri-implant mucositis: The most recent definition of peri-implant mucositis is included within the New Classification of Periodontal and Peri-Implant Diseases and Conditions, 2018 (3). The following definition will be taken as the current definition of peri-implant mucositis in our review: Presence of bleeding and/or suppuration on gentle probing with or without increased probing depth compared to previous examinations and absence of bone loss beyond crestal bone level changes resulting from initial bone remodelling.

Conventional non-surgical treatment of peri-implant diseases: Currently there is no gold standard in the treatment of peri-implant mucositis, several protocols have been described over the years based on the experience of treating gingivitis (4). The treatment is based on the non-surgical removal of plaque deposits and calculus by using plastic or teflon curettes and establishing good plaque control with proper oral hygiene instructions.

Diode laser therapies: There is no consensus on a gold standard protocol for laser treatment for peri-implant diseases. Two types of diode laser therapy will be considered in this review (7,12).

• Photothermal Laser therapy (PT): This therapy is based on the conversion of light energy into thermal energy, increasing the temperature in the tissues and producing injuries that will depend on the degrees reached. Depending on the power at which the laser is used in this therapy, bactericidal, cutting and coagulation effects as well as cellular biostimulation will be obtained (13,14).

• Photodynamic therapy (PDT): Photodynamic therapy is based on a non-thermal photochemical mechanism. A pigment is used, called a photosensitizer, which selectively reaches the cell or microorganism to be eliminated and is irradiated with a wavelength according to the selected pigment. This therapy seeks to obtain bactericidal and bacteriostatic effects (15,16).

-Data analysis 

The articles were compared, and the mean values of the primary variables were directly grouped and analysed using standardised mean difference (SMD) and 95% confidence intervals (CI). All analyses were performed with the IBM® SPSS® Statistics version 21.00 software. Statistical significance was defined for a value of *p* <0.05.

## Results

-Study selection.

As illustrated in the PRISMA flowchart (Fig. 1.), initially, a total of 167 studies were identified across all databases. After the first screening, second screening and bibliographic cross-search, a total of seven studies were included in the present systematic review. Table 1,  Table 1 cont. lists details of the excluded studies (17-24).

**Table 1:**
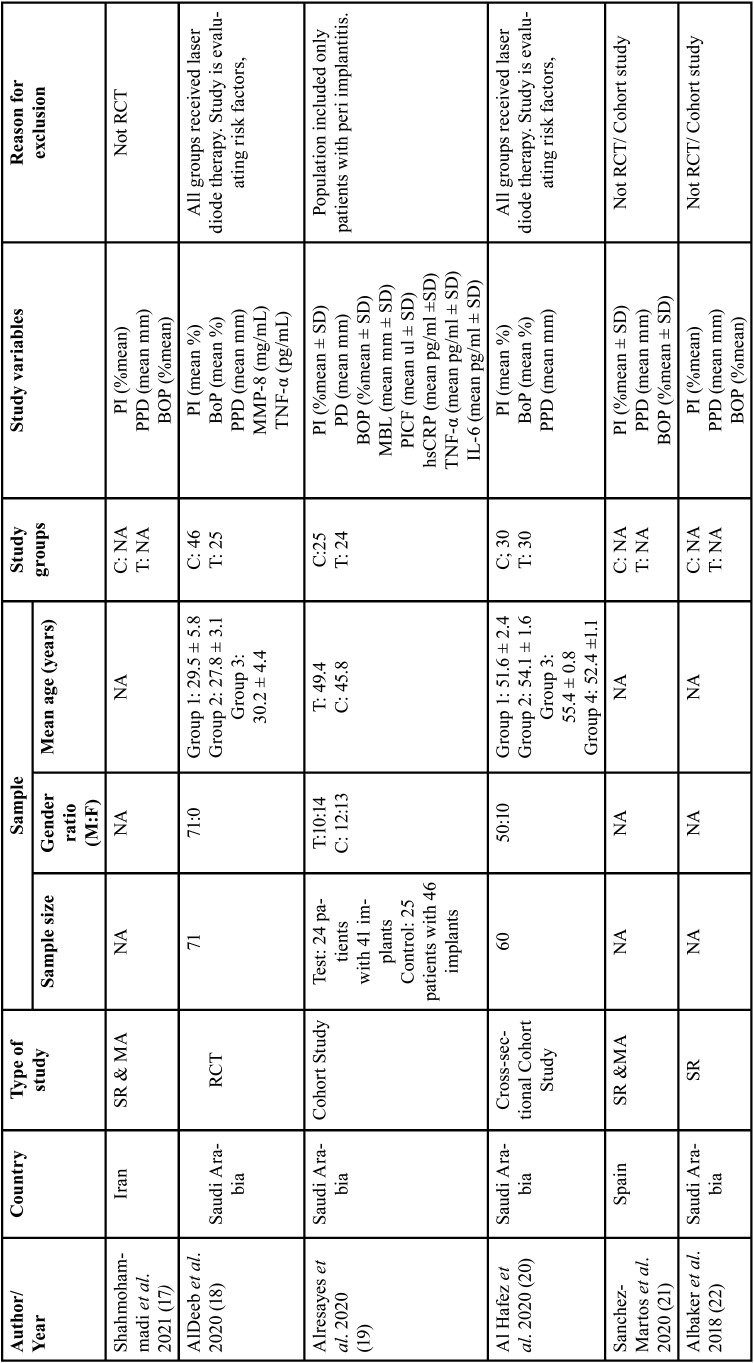
Characteristics of Excluded Studies.

**Table 1 cont.**
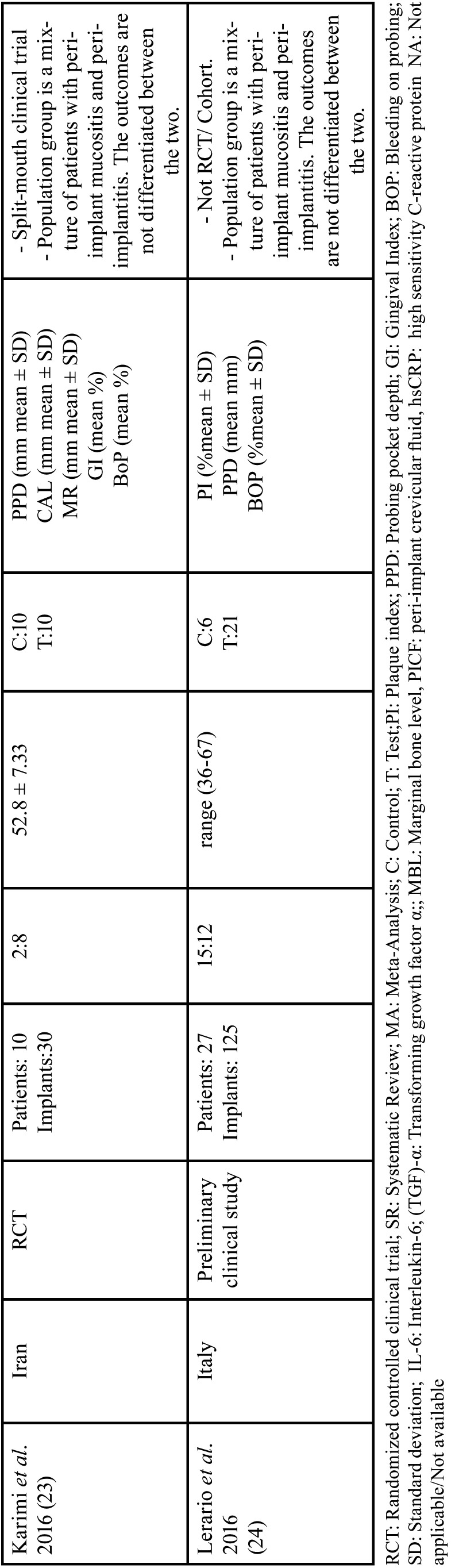
Characteristics of Excluded Studies.

-Characteristics of included studies.

Outlined in Table 2,  Table 2 cont. are the characteristics of the seven studies included (25-31) in the present systematic review. Details of the publication’s author, year, country, type of study, population sample (including sample size, gender ratio and mean age), study groups, follow up time, study variables and risk of bias were listed. All the studies included were randomized controlled trials. The sample size of the population ranged from 38- 220 participants where Al Rifaiy *et al*.’s clinical study (29) had the lowest number of participants while Aimetti *et al*.’s study (27) had the most. When it came to the male-to-female gender ratio, there were generally more male participants. Aimetti *et al*.’s study (27) and Mariani *et al*.’s study (26) were the only studies where there were more female participants. The mean age of the participants ranged from 44.6 years (30) up to 69 years old (29). The number of individuals belonging to the control and test group were generally even for all the studies except for one where there was great a disparity (28). Mariani *et al*.’s (26) had 3 more individuals in the test group compared to the control group. Deeb *et al*.’s study had 30 individuals in the control group and 15 in the test group (28). All the included studies had a follow-up period of 3 months except for Mariani *et al*.’s study that had a longer follow-up period of 12 months (26). All included studies measured bleeding on probing in mean percent. Other study variables included were plaque index, periodontal pocket depth, recession, and levels of MMP-8 and TNF-α. Regarding the risk of bias, five out of the seven studies had a low risk of bias (25-29). The risk of bias was unclear for Javed *et al*’s (31). Al-Sowygh *et al*.’s study, however, had a high risk of bias (30).

**Table 2:**
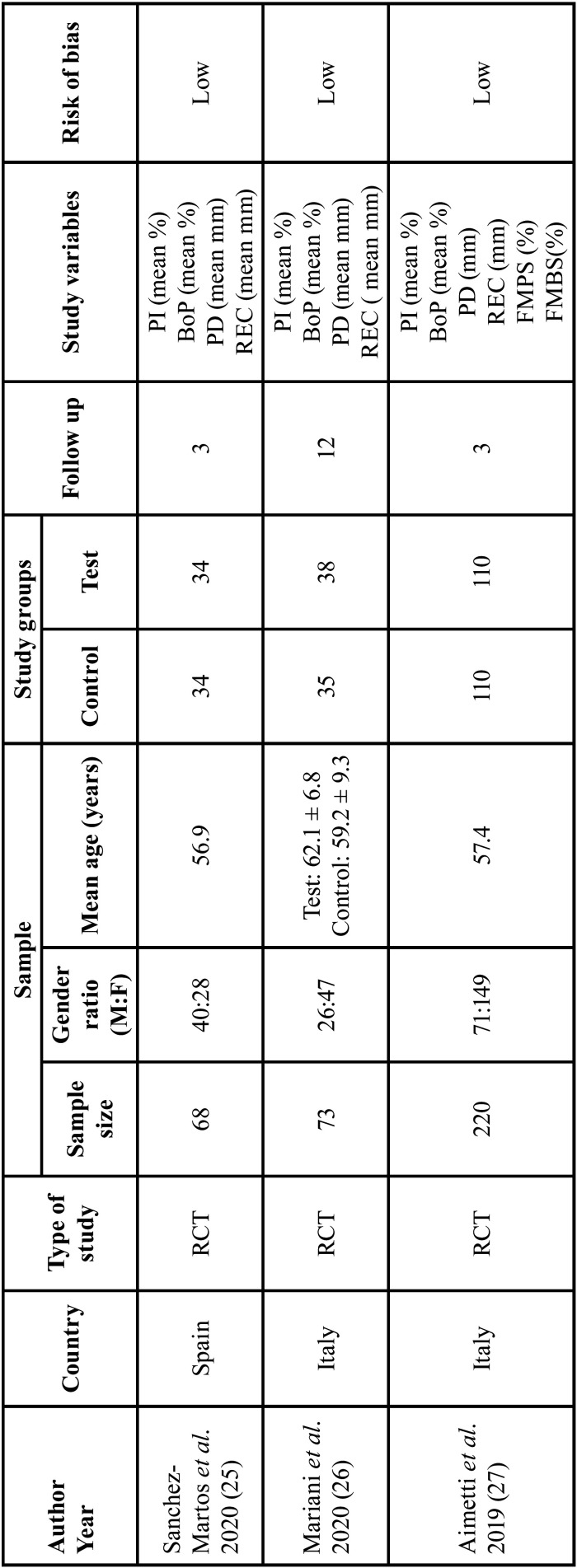
Characteristics of Included Studies.

**Table 2 cont.**
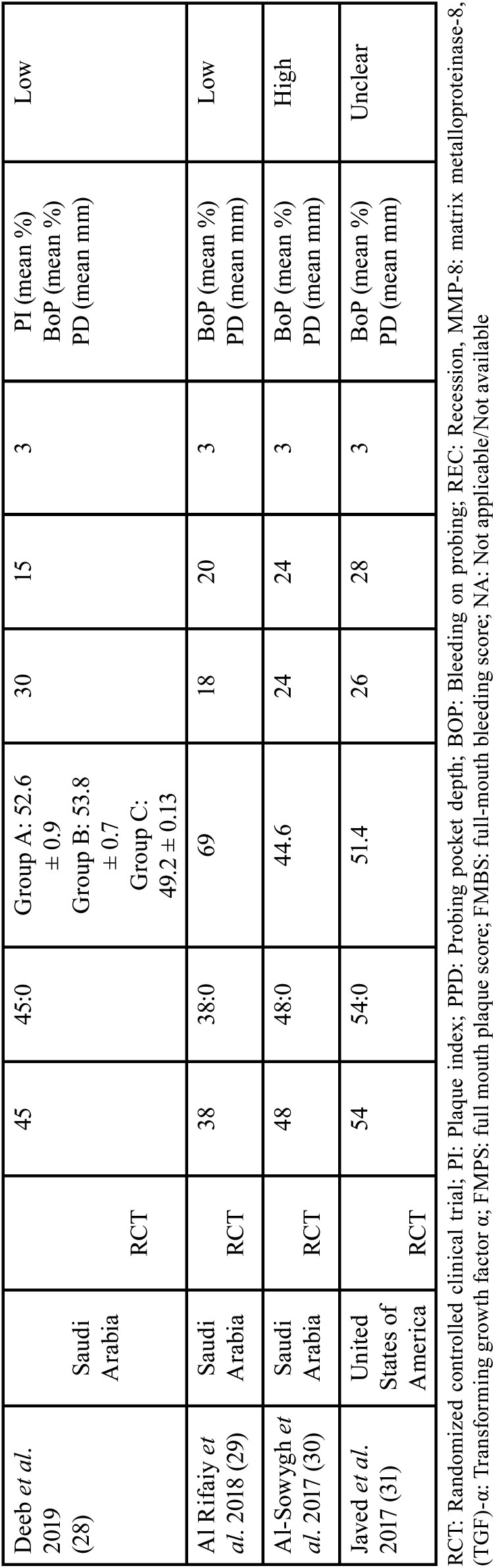
Characteristics of Included Studies.

-Laser and photochemotherapy related parameters.

Table 3 summarizes the technical specifications of the laser therapy used in each study.

**Table 3:**
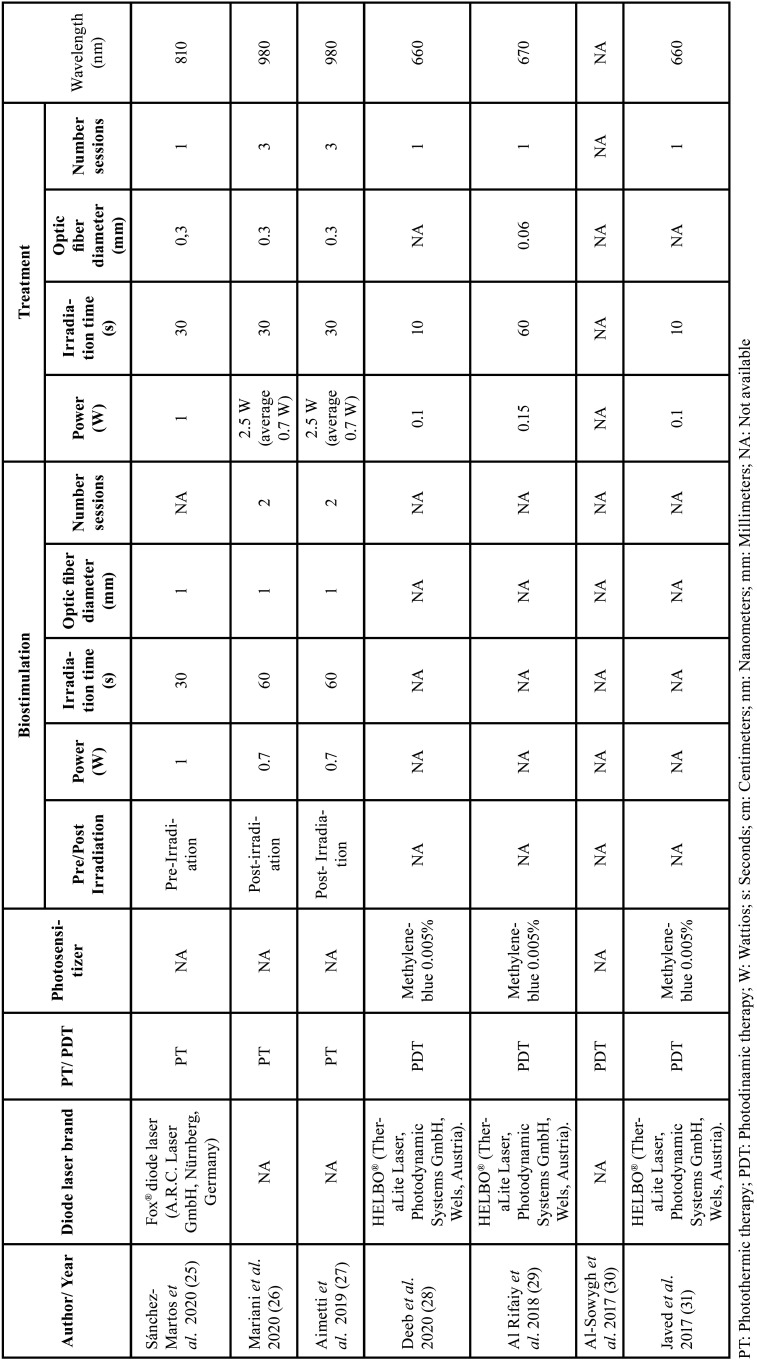
Laser and photosensitizer parameters of included studies.

-Risk of bias across studies 

The risk-of-bias of each study included in the present systematic review was assessed using the Cochrane Collaboration’s tool RoB 2 (11). Illustrated in Fig. 2. is the overall risk-of-bias assessment of each study as well as each domain across all studies. The overall risk-of-bias judgment across all studies for each domain assessed varied. Illustrated in Fig. 2. is a summary of the risk of bias of each factor across all studies based on the judgment of the reviewers.

-Synthesis of the results.

The difference between baseline values and 3-month follow-up values were compared between the control and test groups of each included study (Table 4).

**Table 4:**
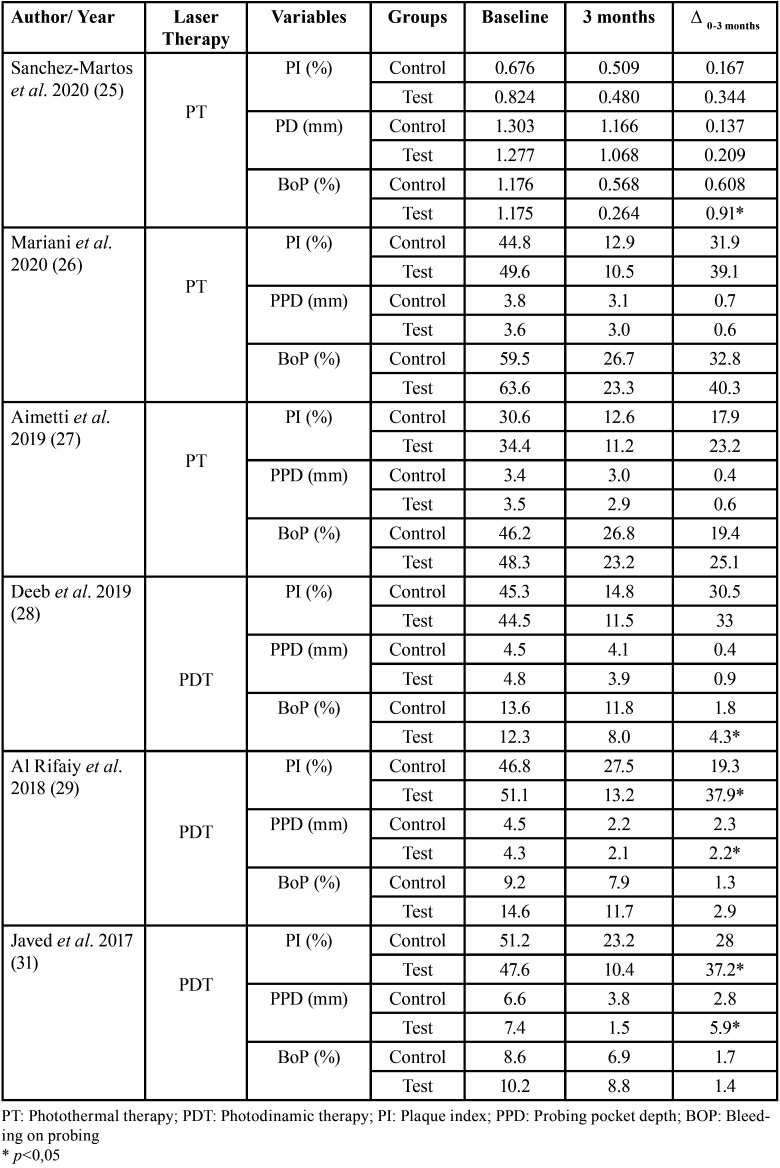
PI, PD and BoP Index at Baseline and 3-month Follow-up Appointment of Included Studies.

Antimicrobial Photodynamic Therapy: In Al Rifaiy *et al*, Javed *et al*., and Deeb *et al*’s aPDT studies yielded a reduction in the plaque index (28,29,31). The reductions seen in the PD over a 3-month period with mechanical debridement alone were 2.3 mm, 2.8mm and 0.4mm accordingly. Two out of the three studies demonstrated a greater reduction in PD in the test group in comparison to the control group. Two out of the three studies demonstrated a greater reduction in BoP in the test group in comparison to the control group (28,31).

Photothermal Laser Therapy: All PT studies demonstrated a PI reduction (25-27). Two out of the three studies demonstrated a greater reduction in PD in the test group in comparison to the control group (25,27). All studies demonstrated a reduction in BoP index.

Discussion

All studies included in the present systematic review are homogenous in terms of the key clinical sign for its diagnosis: BoP. The study population included in the present systematic review is heterogeneous. Some studies test the intervention on a population group of only tobacco consumers. Since tobacco is a crucial risk factor for peri-implant mucositis, the outcome of the intervention will also be affected if the study sample only includes tobacco users. Notably, the main objective of those studies is to determine the efficacy of laser therapy on tobacco users. However, for this present systematic review, using studies that do not have a general representative population makes it difficult to make general conclusive statements.

The aleatory process used is also heterogenous. The stratified block randomization system is notably the best randomization method to implement for the studies included in the present systematic review since this process randomly assigns an equal number of participants to groups and addresses influential characteristics accordingly. It “requires identification of key prognostic characteristics that are measurable at the time of randomization and 50 are considered to be strongly associated with the primary outcome” (32). Therefore, it guarantees a homogenous distribution of participants and eliminates the risk of bias. It is essential to randomize participants well since this is what gives RCTs the level of evidence and prestige when compared to other types of studies. If the sample is already biased from the beginning, the results are difficult to replicate and therefore there is a lack of confidence in the studies

Clinically, the follow-up period is relevant since it provides evidence of treatment efficacy, the duration of the effect and the level of compliance of the 51 participants in the maintenance phase (33). Measuring the clinical parameters only after months have elapsed is difficult to determine the course of action of the intervention. Longer follow-up period would have been useful to see if there were any pathogenic bacteria regrowth and the advancement of peri-implant mucositis into periimplantitis despite the patient undergoing laser therapy (30). On the other hand, it is also important to note that at this stage, it is mostly the patient’s responsibility to maintain a low pathogenic bacterial load through oral hygiene habits and eliminating risk factors (33).

-Plaque index 

The included studies demonstrate the benefits of both PT and aPDT as an adjuvant to mechanical debridement in reducing the overall plaque index. Table 4 highlights the significant reduction of plaque after both types of laser therapies over a period of 3 months. It is, however, clear that the reduction seen in aPDT surpassed that seen in PT. This may be because the outcome variables are dependent on the initial baseline value. Initially, if a patient has a remarkably high PI, after mechanical and chemical cleaning, the changes observed will be more drastic in comparison to a case when the patient initially has very minimal plaque. Similar correlations were mentioned in a similar systematic review (21). Additionally, the decrease in the plaque scores seen with both interventions may be due the laser therapy itself. aPDT promotes an antimicrobial environment by rapidly selecting and destroying targeted bacterial species, inactivating virulence-associate protease and detrimental host factors (34). The photosensitizers are also able to flow deeply into the sulcus and thus maximize the effects of aPDT (34,35). It is also possible that oral hygiene maintenance has improved over the course of the clinical trial. Hence, the reduction of the plaque index seen in both diode laser therapies can be thanks to mechanical debridement, cooperation of the participants and very minimally, the laser therapy itself.

-Probing pocket depth

Regarding the probing depth, the peri-implant pocket depth may vary greatly but it helps detect the presence of inflammation. Amongst the clinical trials implementing PT, the variations between the control and the test groups range from 0.072- 0.2 mm. In addition to these variations being very minute, the values are not statistically significant and therefore the variations seem more likely due to other factors unrelated to the intervention such as the method of mechanical debridement, oral hygiene habits and lifestyle choices of the participants or chance. Amongst the clinical trials implementing aPDT, the variations between the control and test groups range from 0.1- 3.1 mm. The greater statistically significant variations observed may be due to variations in initial baseline values, photosensitizer placement methodology and patient compliance. As noted earlier, baseline values are in determining the changes seen at the follow-up appointment. The average baseline values for Javed *et al*’s study (31) was 7.4mm- the highest amongst all studies. 3 months after aPDT laser diode therapy, the probing pocket depth was reduced by 5.9mm. This is the highest reduction seen among all clinical trials. Although their results are statistically significant and therefore the difference seen is more likely due to the intervention employed and not by chance, it is important to note that the drastic decrease in probing pocket depth seen in this study is most likely due to the large initial probing depth. It is also important to note that the decrease in probing pocket depth seen in these studies may simply be due to the improvement of the plaque index. There is a direct relationship between plaque accumulation and soft tissue inflammation. For this reason, it is safe to conclude that an improved plaque index also results in a decrease in inflammation and therefore probing depth. Further research is needed to consider all these factors in order to factually state the specific benefits of the laser in terms of probing depth.

-Bleeding on probing

**Figure 3:**
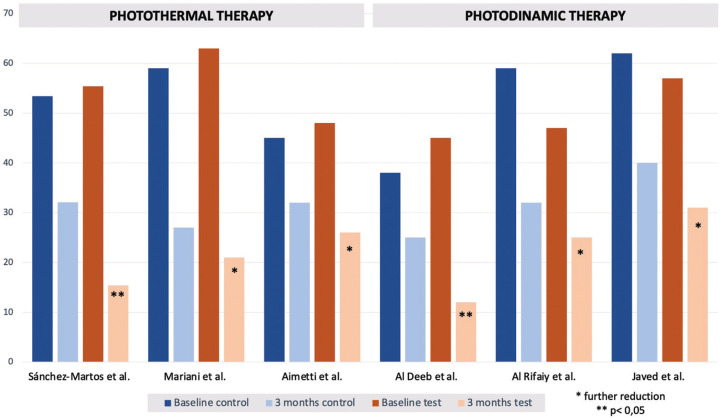
Graphical representation of the changes in bleeding on Probing Index 3 months after laser 5 therapy.

With regards to the bleeding on probing index, although the definitions of peri-implant mucositis had differed over the years, what has remained persistent is that the key diagnostic clinical manifestation of peri-implant mucositis is bleeding on gentle probing. Bleeding on gentle probing has a low positive predictive value but a high negative one. In essence, implants have a higher tendency to bleed than natural teeth due to their higher risk for early inflammation and longer healing period time (12,36). Therefore, an implant that bleeds does not bring significant value compared to an implant that does not bleed. For that reason, the efficacy of both types of therapies will be dependent on the results obtained from this clinical parameter. As depicted in Fig. 3, PT resulted in a greater significant change in the BoP index in comparison to the aPDT. Regarding PT, the reductions seen in the bleeding on probing over a 3-month period were 0.911% (25), 25.1% (27) and 40.3% (26). The reductions seen in the bleeding on probing over a 3-month period when aPDT was used were 2.9% (29), 1.4% (31) and 4.3% (28). The statistical significance of the results obtained for photothermal studies was only statistically significant during the 3-month follow-up for Sanchez-Martos *et al*’s trial (25). This may be due to the variation of baseline characteristics of the participants of each study. In Aimetti *et al*.’s (27) and Mariani *et al*’s studies (26), a considerable number of participants has a history of treated periodontitis. PT resulted in a greater significant change in the BoP index in comparison to aPDT. If the sample population is composed of patients who had a history of periodontal disease, one can conclude that the microbial composition, as well as the oral hygiene habits, may influence the results (37). Al Rifaiy *et al*. (29) and Javed *et al*.’s (31) studies showed that the intervention had no effect. Their studies compared the efficacy of the therapies in e-cigarette smokers and tobacco smokers. Vasoconstriction due to smoking, whether tobacco smoking or e-cigarette smoke, can be seen due to the pathophysiological mechanism of nicotine. In addition to reduced cellular healing ability, nicotine has also been reported to reduce the tendency of bleeding (38,39). Hence, if smokers continue to consume nicotine throughout the clinical trial, the decrease in blood flow in gingival blood vessels results in lower BoP scores regardless of the intervention used. One of the main benefits of diode laser therapy is photobiomodulation. Photobiomodulation is one of the most important aspects of photothermal laser therapy that has yet to be studied. In laser therapy, it is the promotion of cellular regeneration in the deepest layers of soft tissue through the use of a laser. Essentially, lasers promote and activate cellular proliferation, collagen synthesis, mitochondrial respiration and ATP synthesis (36). LLLT have a photobiomodulating effect that promotes wound healing and reduces inflammation. This results in the possibility of the BoP index to decrease drastically, from 100% to 43%, at a 2-year follow-up of patients with peri-implantitis, for example. A sTable peri-implant tissue allows the anti-inflammatory effect to remain for longer periods of time and generally improves its health and therefore clinical parameters.

The limitations of the present systematic review include the fact the number of studies and follow-up period is limited. They also have a variety of risk-of-bias. For that reason, although the aPDT seems to show more promising results in terms of the reduction of some clinical parameter signs, one must take into consideration that those studies showed an overall higher risk of bias compared to the studies experimenting with PT. Future researchers should therefore consider carrying out more studies, specifically, RCTs where there is adequate and non-bias randomization and a larger sample population size. Therefore, it can be concluded that conclusions are difficult to generalize due to the heterogeneity in the methodology of the included studies. However, this systematic review suggests that aPDT alongside mechanical debridement demonstrated greater improvements in the PI and PD. Other factors besides the laser therapy itself may account for these findings. As for BoP index, PT demonstrated greater improvements due to its photo-biomodulating effects. Future research should be guided towards determining whether one therapy is more useful in specific populations or clinical situations.
